# Editorial: Artificial intelligence in brain-computer interfaces and neuroimaging for neuromodulation and neurofeedback

**DOI:** 10.3389/fnins.2022.974269

**Published:** 2022-07-15

**Authors:** Hiram Ponce, Lourdes Martínez-Villaseñor, Yinong Chen

**Affiliations:** ^1^Facultad de Ingeniería, Universidad Panamericana, Ciudad de Mexico, Mexico; ^2^Barrett Honors Faculty, School of Computing and Augmented Intelligence, Arizona State University, Tempe, AZ, United States

**Keywords:** artificial intelligence, neuroimaging, neuromodulation, neurofeedback, brain-computer interface

## 1. Introduction

Neuromodulation and neurofeedback are two alternative non-pharmacological ways of treating neurological related diseases and disorders (Grazzi et al., 2021; Hamed et al., 2022). Neuromodulation refers to as the modulation of brain function *via* the application of weak direct current (Lewis et al., 2016). Neurofeedback is a psychophysiological procedure that provides models of neural activity to subjects aiming to control them online (Marzbani et al., 2016). Both alternatives have been successfully applied in a variety of neurological conditions including Parkinson's disease, chronic pain, epilepsy, depression, essential tremor, among many others (Tsatali et al., 2019; Baptista et al., 2020; Hamed et al., 2022). Typical challenges in these types of treatment are related to the way of collecting data, the improvement in the efficiency of the methods, the interpretability of feedback signals, to name a few (Johnson et al., 2013; Lewis et al., 2016; Marzbani et al., 2016; Papo, 2019).

Currently, artificial intelligence (AI), and more particular machine learning (ML), allow a better understanding of brain activity and a better brain-computer interface (BCI) mechanism of interaction (Zhang et al., 2020). The integration of AI/ML in the data collection and monitoring phases of neuromodulation or neurofeedback can be used for early diagnosis and accurate non-pharmacological treatment of neurological diseases and disorders. ML enables analyzing large volumes of patient information to improve the efficiency of neuromodulation and neurofeedback. The introduction of AI/ML in BCI and neuroimaging empowers these techniques for data acquisition, monitoring, analysis and prevention of neurological diseases and disorders (Patel et al., 2021). Thus, further investigation is necessary to understand and extend brain-computer interfaces and neuroimaging techniques implementing AI/ML. The latter integration into neuromodulation or neurofeedback can provide new opportunities to improve significantly the efficiency in the output response of these types of treatment for neurological diseases and disorders.

This Research Topic aims to cover studies at the intersection of AI/ML-based BCI and neuroimaging with neuromodulation and neurofeedback in the treatment of neurological diseases and disorders. We received five submissions of which four were published: two reviews and two original research articles.

First, Lopez-Bernal et al. present a survey that provides an insight into the basics behind electroencephalogram (EEG)-based BCI systems directed toward imagined speech recognition. The authors reviewed the most relevant and recent studies with the aim of finding the most commonly used methods and techniques on pre-processing, feature extraction, and classification tasks. They identified trends and challenges to achieve a practical application of EEG-based BCI systems toward imagined speech decoding.

Another important overview was presented by Olsen et al. reviewing the movement-related cortical potential (MRCP) literature related to ecologically valid movements tasks. The ecological validity discusses the generalizability of the findings to real-word situations. Their findings suggest that some studies demonstrated differences in MRCP features in populations of older adults and patient with Parkinson's disease. Signals appear to vary across different movement tasks. Research is largely done in healthy populations, hence, further research in MRCP is needed in populations with neurological and age-related conditions.

Affiliative feelings and complex emotions are important for mental health and the EEG is a suitable tool for therapeutic application in the clinical environment. De Filippi et al. proposed a method to classify discrete complex emotions. EEG-based affective computing studies commonly use passive elicitation through single-modality stimuli, the authors integrated passive and active elicitation methods. Their proof of concept shows evidence that anguish and tenderness present distinct electrophysiological correlates that can be identified using a Support Vector Machine classifier. This paper's contribution in biofeedback and non-invasive neuroimaging approaches may help in restoring balanced neural activity in people with emotional disturbances.

Finally, Rojas et al. proposed a sensing platform that can help patients with mobility impairments to manipulate electronic devices in order to increase their independence. They used three hands-free signals as input, voice commands, head movements, and eye gestures, in the sensing scheme. The signals were recollected from non-invasive sensors: a microphone, an accelerometer, and an infrared oculography. Two volunteers with severe disabilities performed 15 common skills to wheelchair users for evaluation. Their results showed high performance developing head movement skills, hence volunteers had trouble with voice control skills. Multiple applications can be developed to help people with disabilities with the use of everyday devices.

These contributions provide the perspectives and trends in the intersection of AI/ML-based BCI and neuroimaging with neuromodulation and neurofeedback in the treatment of neurological diseases and disorders.

## Author contributions

HP, LM-V, and YC contributed to manuscript writing, revision, read, and approved the submitted version. All authors contributed to the article and approved the submitted version.

## Conflict of interest

The authors declare that the research was conducted in the absence of any commercial or financial relationships that could be construed as a potential conflict of interest.

## Publisher's note

All claims expressed in this article are solely those of the authors and do not necessarily represent those of their affiliated organizations, or those of the publisher, the editors and the reviewers. Any product that may be evaluated in this article, or claim that may be made by its manufacturer, is not guaranteed or endorsed by the publisher.
